# SimHap GUI: An intuitive graphical user interface for genetic association analysis

**DOI:** 10.1186/1471-2105-9-557

**Published:** 2008-12-25

**Authors:** Kim W Carter, Pamela A McCaskie, Lyle J Palmer

**Affiliations:** 1 Western Australian Institute for Medical Research and UWA Centre for Medical Research, University of Western Australia, Perth, Australia; 2 School of Mathematics and Statistics, University of Western Australia, Perth, Australia; 3 Centre for Genetic Epidemiology and Biostatistics, University of Western Australia, Perth, Australia; 4 Telethon Institute for Child Health Research, UWA Centre for Child Health Research, University of Western Australia, 100 Roberts Rd, Subiaco, Western Australia 6008, Australia

## Abstract

**Background:**

Researchers wishing to conduct genetic association analysis involving single nucleotide polymorphisms (SNPs) or haplotypes are often confronted with the lack of user-friendly graphical analysis tools, requiring sophisticated statistical and informatics expertise to perform relatively straightforward tasks. Tools, such as the *SimHap *package for the R statistics language, provide the necessary statistical operations to conduct sophisticated genetic analysis, but lacks a graphical user interface that allows anyone but a professional statistician to effectively utilise the tool.

**Results:**

We have developed SimHap GUI, a cross-platform integrated graphical analysis tool for conducting epidemiological, single SNP and haplotype-based association analysis. SimHap GUI features a novel workflow interface that guides the user through each logical step of the analysis process, making it accessible to both novice and advanced users. This tool provides a seamless interface to the *SimHap *R package, while providing enhanced functionality such as sophisticated data checking, automated data conversion, and real-time estimations of haplotype simulation progress.

**Conclusion:**

SimHap GUI provides a novel, easy-to-use, cross-platform solution for conducting a range of genetic and non-genetic association analyses. This provides a free alternative to commercial statistics packages that is specifically designed for genetic association analysis.

## Background

While the growth in the volume of genetic data available has led to many new discoveries, it is becoming increasingly important to find ways in which to easily analyse large of volumes of data. This is certainly the case with genetic association studies, where high-throughput genotyping technologies have brought about the potential for hundreds of thousands of data points per individual subject [1].

A graphical user interface (GUI) is still a rare feature amongst currently available genetic analysis packages, particularly those used to analyse single nucleotide polymorphisms (SNPs) or haplotypes. A well designed user interface would allow users without a comprehensive knowledge of statistical modelling or command line operation to perform complex analyses.

Commercially available statistics software packages, such as SPSS (SPSS Inc., 2008) and Stata (StataCorp. 2008), may be useful, but are not specifically designed to analyse genetic data, requiring sophisticated prior knowledge for the end-user. Another major annoyance is the lack of integration between statistical and analytical packages [2], often with one program required for epidemiological analysis, a separate program for SNP analysis, and a third used for haplotype analysis.

*SimHap *[3] is a statistical analysis package for genetic association testing, available in R [4], which amongst other features, infers haplotypes for unrelated individuals with unknown phase. Although various programs currently exist for haplotype analysis, *SimHap *is unique in a number of ways. It uses a multiple-imputation (MI) based approach to test for association, which incorporates information about uncertainty around inferred haplotypes. This approach uses current expectation maximisation (EM) methods for the estimation of haplotype frequencies from unphased genotype data [5]. To utilize the posterior distribution of diplotype (a haplotype pair) probabilities, the MI approach of Rubin [6] was implemented, where a series of "complete" data sets are generated containing all data from the original set as well as additional dummy variables for each haplotype, the values of which indicate the number of copies of that haplotype observed in an individual's diplotype (0, 1 or 2). For individuals with known phase (only one diplotype), the values for these haplotype variables remain constant for each of the generated data sets. For individuals with ambiguous phase, their haplotype values will be sampled from their predictive distribution, containing only those diplotypes consistent with their genotypes. This is a novel approach that provides an empirical distribution of the haplotypic effects and their significance levels.

We have developed SimHap GUI as an intuitive graphical tool for conducting genetic association analysis. At its core, SimHap GUI utilises the *SimHap *R package and the R statistical language. SimHap GUI is a novel custom-designed integrated tool for conducting epidemiological, single SNP and haplotype-based association analyses within a single application, and provides a free alternative to commercially available statistics packages.

## Results and discussion

### Implementation

SimHap GUI is written in Java (requires Java 1.5+) and will operate on platforms where Java is available. This tool has been successfully tested on Windows, Linux and MacOS operating systems. SimHap GUI requires an installation of the R statistics lanuguage (2.4.0+) and an installation of the SimHap R package. This tool runs optimally on a computer with a monitor resolution of 1024 × 768, at least 128 Mb of RAM and a Pentium 4+ CPU. SimHap GUI has been successfully operated on datasets with thousands of individuals, hundreds of phenotype variables, and thousands of SNPs. SimHap GUI is generally only limited by the amount of system memory available to Java.

The SimHap GUI interface is written in Java Swing, and uses the Synthetica look-and-feel suite [7] to enhance the useability and functionality of the interface (compared with standard Swing interfaces). We have also utilised the Swing Worker [8] library, which provides a mechanism for providing updates to the user interface while running long analytical tasks, such as performing thousands of haplotype simulations. Both Synthetica and Swing Worker are provided with the SimHap GUI installation. SimHap GUI is provided as a single cross-platform installer, using the IzPack [9] packaging system, which provides a simple standardised graphical installer tool that both technical and non-technical users will be comfortable with.

### Graphical User Interface (GUI)

SimHap GUI allows the user to conduct association analysis of binary, quantitative, longitudinal and survival (right-censored) outcomes using phenotypic data, and genetic SNP data and haplotype data, in unrelated individuals.

One key feature of SimHap GUI is the workflow interface, which guides the user through each logical step of the analytical process. This workflow concept is central to providing an intuitive user interface accessible to both novice and advanced users.

The user initially selects a standard comma separated value (CSV) file containing phenotypic information for a set of individuals (one row of data per person), as can be obtained from most spreadsheet and statistics software. The user also selects a CSV file containing genotypes for a series of SNP markers for the same individuals (not required for non-genetic modelling), and selects the character(s) signifying missing data in the input files. SimHap GUI examines the input files to ensure correct formatting, completeness, and the correct corresponding individual identifier between phenotype and genotype files. Genotype files are examined to ensure biallelic SNPs are input, where the user is given the option to remove multi-allelic markers. Once data checking is complete, the user can choose to perform epidemiological modelling (without genetic markers), single SNP association analysis, or haplotype association analysis. Users are guided through each of these analytical tasks in a straight-forward series of steps, with a standardised model building screen central to each of the analysis types.

**Figure 1:**
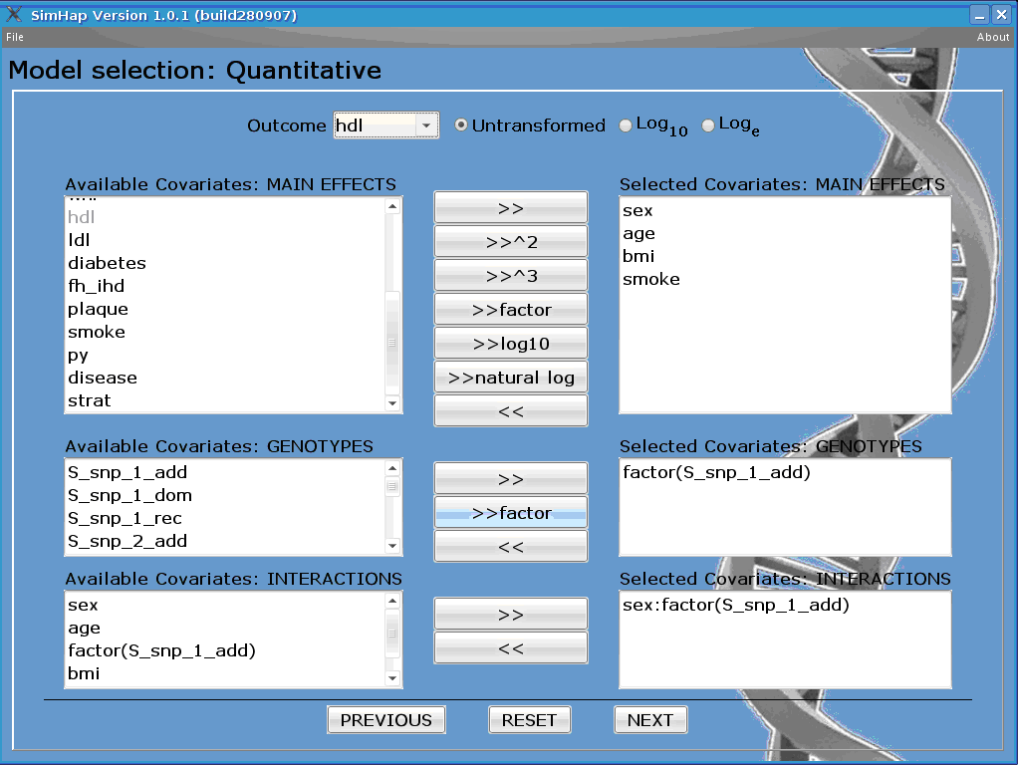
**Example SimHap GUI model building interface**.

Figure 1 is an example of the model building screen for a single SNP analysis with a quantitative outcome using SimHap GUI. At the top of the screen, *hdl *(cholesterol) has been selected as the outcome of interest, with the outcome normally distributed (*Untransformed*). Log base 10 and natural log of the outcome are available to transform non-Normally distributed outcomes. In the *MAIN EFFECTS *section are the available and selected covariates for this model, namely *sex*, *age*, *bmi *and *smoke*. Covariates can also be added as squared or cubic terms, logged (base 10 or natural log), and as factors (for categorical terms). In the *GENOTYPES *section are the available and selected SNPs to be analysed in the model. SNP covariates are denoted with the *S_ *prefix, while the *_add*, *_dom *and *_rec *terms refer to analysing the SNP under an additive, dominant or recessive genetic model. SNPs can also be analysed under a codominant model by adding the SNP as a *factor*. In the *INTERACTIONS *section are available and selected covariate terms to be analysed for statistical interactions; in this case, an interaction between *sex *and *SNP_1 *under a codominant model. Additional files 1, 2, 3, 4, 5, 6, 7, 8 provide a graphical representation of each of the phases of analysis for an example single SNP analysis. The SimHap GUI software manual also provides a detailed description of the analysis process.

### Case Studies

SimHap GUI, and its earlier Beta 1 and Beta 2.1 releases, have been extensively utilised in a range of genetics projects recently published.

In the area of cancer research, SimHap GUI has been used in studies such as Sak et al [10], to examine the association between polymorphisms in the *XPC *gene and bladder cancer susceptibility. Choudhury et al [11] also examined haplotypes of DNA repair proteins to find genetic variants that may modulate predisposition to bladder cancer.

SimHap GUI has been used extensively in the field of cardiovascular disease genetics. Several studies has used this tool to examine SNP and haplotype effects of genes related to abdominal aortic aneurysm [12-14]. Studies by both Horne et al [15] and McCaskie et al [16] have used SimHap GUI to investigate the association between genetic variation in the cholesteryl ester transfer protein gene and cardiovascular disease. SimHap GUI has also been used to investigate SNP and haplotype associations with metabolic syndrome [17-20] and atherosclerosis [21-24] related outcomes.

In the area of genetic epidemiology related to the Mendelian Randomization (MR) technique, a number of groups have utilised SimHap GUI. Brunner and colleagues [25] used SimHap GUI to generate haplotypes for three tagging polymorphisms from the C-reactive protein (*CRP*) gene in a study of 5,274 men and women. Studies by Lawlor et al [26] and Kivimaki et al [27] similarly this software for analysis of CRP mutations using MR.

Other diverse studies include the use of SimHap GUI to investigate genetic influences of the melanocortin 1 receptor with sensitivity to photochemotherapy [28], polymorphisms within the macrophage migration inhibitory factor with relation to acute lung injury in patients with sepsis [29], associations between cytokine polymorphisms and outcomes after renal transplantation [30], and genetic predictors for the development of microalbuminuria in children with type 1 diabetes [31].

The wide range of example publications described here highlights the significance of the SimHap GUI software providing an easy-to-use powerful interface for both novice and advanced genetic association analyses.

### GUI versus R package

One of the critical distinctions to make with the SimHap GUI software is the difference between the *SimHap *R package, and the Java based interface described in this manuscript. The backend *SimHap *R package simply provides the statistical operations to conduct particular analytical tasks, with the onus on the user to have technical knowledge of the statistical methods being employed and expertise with the command line interface of the R language. Users who are not professional statisticians may be discouraged by the difficulty of operating under a command-line interface.

The SimHap GUI interface provides the functionality, accessibility and the guided analytical approach that cannot be found in the command line package. The user interface is designed around the premise of a workflow analysis model, which mimics the logical analytical processes required to conduct a particular statistical test. This user-friendly, intuitive interface has been designed to satisfy the needs of both the technical and non-technical statistical user, and does not require sophisticated informatics knowledge to operate. Using the novel model building interface, users can perform tasks ranging from simple univariate linear modelling, through to more sophisticated tasks such as multivariate modelling of longitudinal outcomes with gene:gene and gene:environment interactions. A standardised interface is provide for users to conduct epidemiological (no genetics factors), single SNP and haplotype association analyses.

Features of SimHap GUI that are not provided in the *SimHap *R package include: an intuitive GUI for model building and guiding the overall analysis process; sophisticated data checking of phenotype and genotype data; automatic conversion of data for single SNP and haplotype association analysis; automatic calculation of allele frequencies and genotype distribution; quantile-quantile plotting for Normality of quantitative traits; and real-time estimation of the haplotype imputation simulation progress. SimHap GUI implements all of the functions from the *SimHap *R package.

## Conclusion

In summary, SimHap GUI provides a cross-platform, intuitive and integrated interface for conducting a range of genetic and non-genetic association analyses.

## Availability and requirements

- **Project name**: SimHap GUI

- **Project home page**: 

- **Operating system(s)**: Platform independent (tested on Windows, Linux and MacOS)

- **Programming language**: Java

- **Other requirements**: Java 1.5+; R 2.4.0+ (available from ); *SimHap *R package from CRAN (available from )

- **Licence**: Free for non-commercial use

## Authors' contributions

KWC designed and developed the Java GUI interface. PAM assisted with integration of statistical methods and aided with design of the GUI. LJP supervised the design and coordinated the development of the software.

## Supplementary Material

Additional file 1 **SimHap GUI file selection screen.** This screenshot shows the selection of phenotype and genotype CSV files for analysis in SimHap GUI. Click here for file

Additional file 2 **SimHap GUI input parameter selection screen.** Following selection of input files, this screenshot shows the user specifying input parameters, and a summary of the input data file characteristics. Click here for file

Additional file 3 **SimHap GUI major allele selection screen.** After the user has selected to perform a 'single SNP' analysis, the user can specify the major allele for polymorphism in the input genotype file (as illustrated in this screenshot). Click here for file

Additional file 4 **SimHap GUI normality plots.** This screenshot shows the user checking whether quantitative variables to be analysed are normally distributed. This screen option is available when the user is ready to select a particular type of outcome (binary, quantitative, longitudinal and right-censored) for analysis. Click here for file

Additional file 5 **SimHap GUI model building screen for single SNP analysis.** This screenshot shows the model building screen in SimHap GUI, where the user has selected to analyse a quantitative outcome (*HDL*), and has selected various covariates (*SEX*, *AGE*, *BMI*, *SMOKE*) and a polymorphism of interest (*SNP1*). Click here for file

Additional file 6 **SimHap GUI model parameters.** This screenshot shows the display presented after the model building screen, where the user can specify additional subset parameters, and other statistical parameters. Click here for file

Additional file 7 **SimHap GUI results summary.** After the user has built their desired statistical model, SimHap GUI runs the analysis, and the summary results are presented as illustrated in this screenshot. Statistically significant results are highlighted in red for easy identification. Click here for file

Additional file 8 **SimHap GUI detailed results summary.** The screenshot shows the detailed statistical information provided, in addition to the summary statistics described in the previous figure. For example, marginal means by genotype group are provided in this detailed summary. Click here for file
