# Supplementary figures and images for: SimHap GUI: An intuitive graphical user interface for genetic association analysis
Source: BMC Bioinformatics. 2008 Dec 25;9:557. doi: 10.1186/1471-2105-9-557 (PMC2639440; doi:10.1186/1471-2105-9-557)

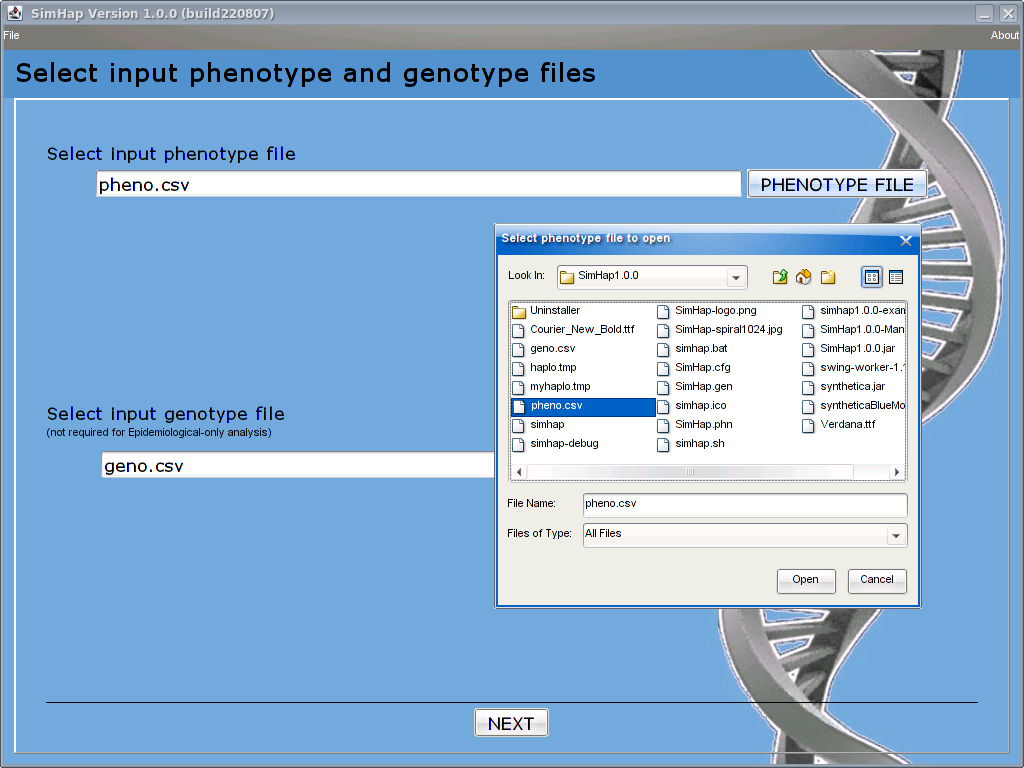

Supplement: Additional file 1 — SimHap GUI file selection screen. This screenshot shows the selection of phenotype and genotype CSV files for analysis in SimHap GUI. [file 1471-2105-9-557-S1.png]

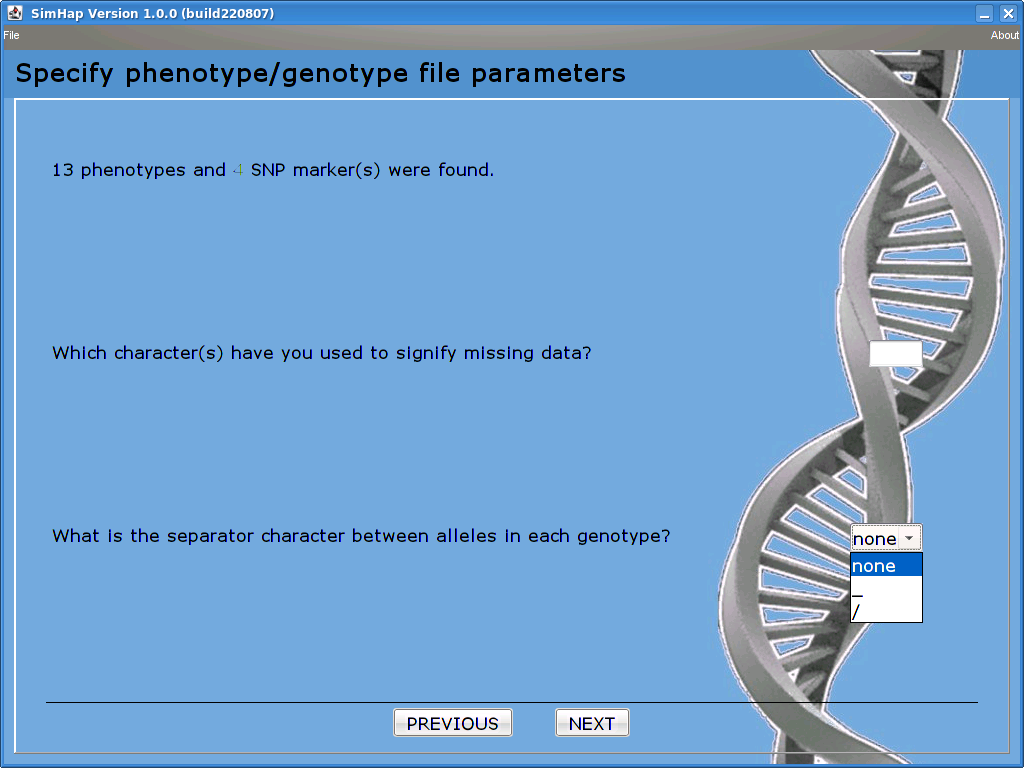

Supplement: Additional file 2 — SimHap GUI input parameter selection screen. Following selection of input files, this screenshot shows the user specifying input parameters, and a summary of the input data file characteristics. [file 1471-2105-9-557-S2.png]

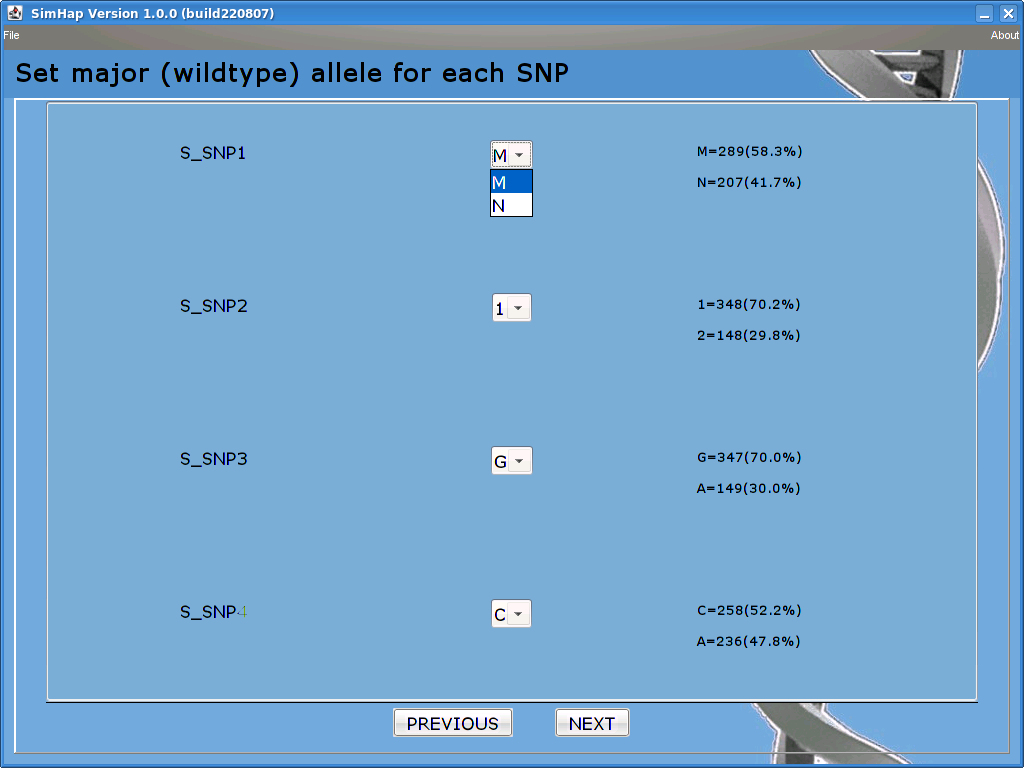

Supplement: Additional file 3 — SimHap GUI major allele selection screen. After the user has selected to perform a 'single SNP' analysis, the user can specify the major allele for polymorphism in the input genotype file (as illustrated in this screenshot). [file 1471-2105-9-557-S3.png]

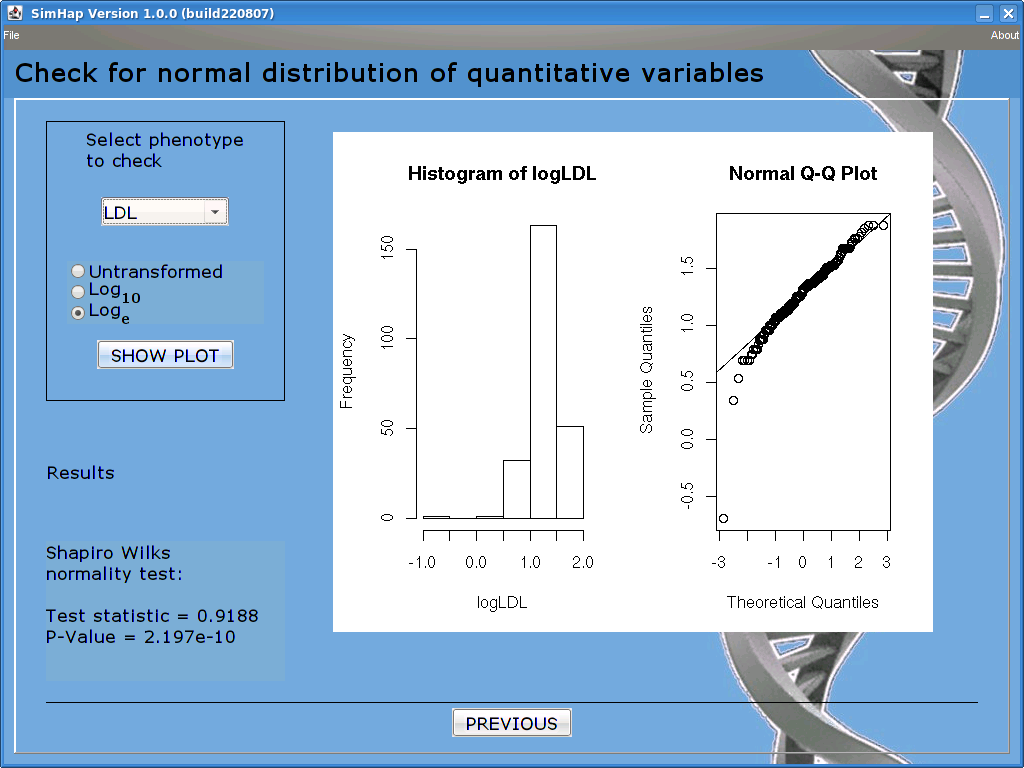

Supplement: Additional file 4 — SimHap GUI normality plots. This screenshot shows the user checking whether quantitative variables to be analysed are normally distributed. This screen option is available when the user is ready to select a particular type of outcome (binary, quantitative, longitudinal and right-censored) for analysis. [file 1471-2105-9-557-S4.png]

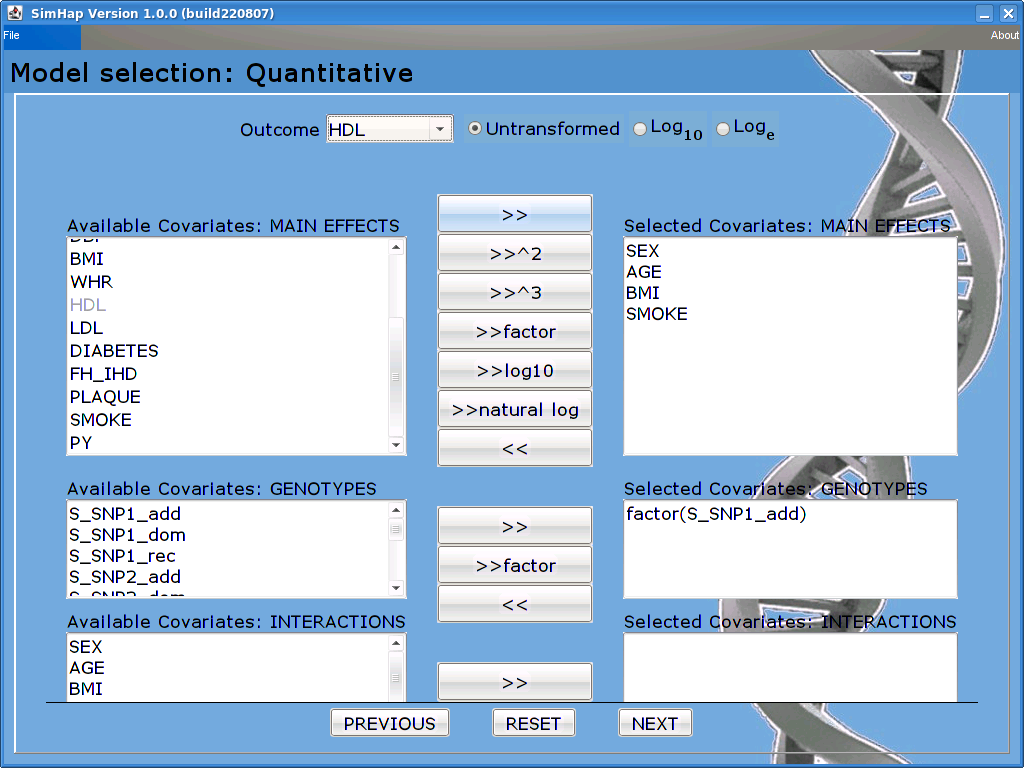

Supplement: Additional file 5 — SimHap GUI model building screen for single SNP analysis. This screenshot shows the model building screen in SimHap GUI, where the user has selected to analyse a quantitative outcome (HDL), and has selected various covariates (SEX, AGE, BMI, SMOKE) and a polymorphism of interest (SNP1). [file 1471-2105-9-557-S5.png]

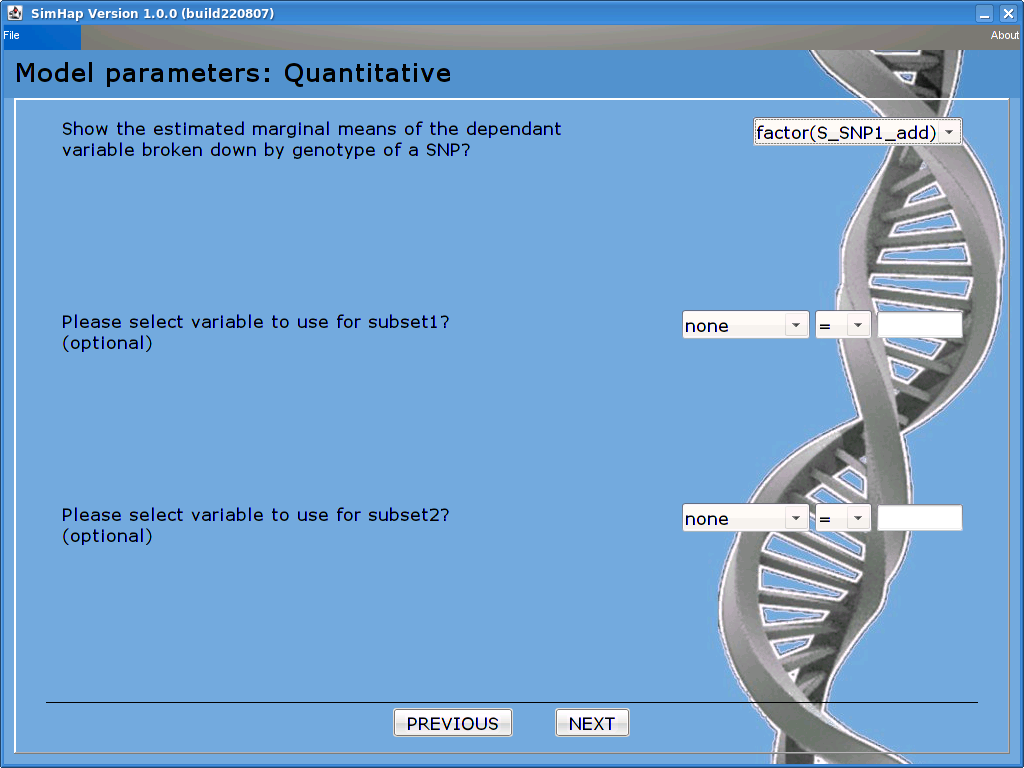

Supplement: Additional file 6 — SimHap GUI model parameters. This screenshot shows the display presented after the model building screen, where the user can specify additional subset parameters, and other statistical parameters. [file 1471-2105-9-557-S6.png]

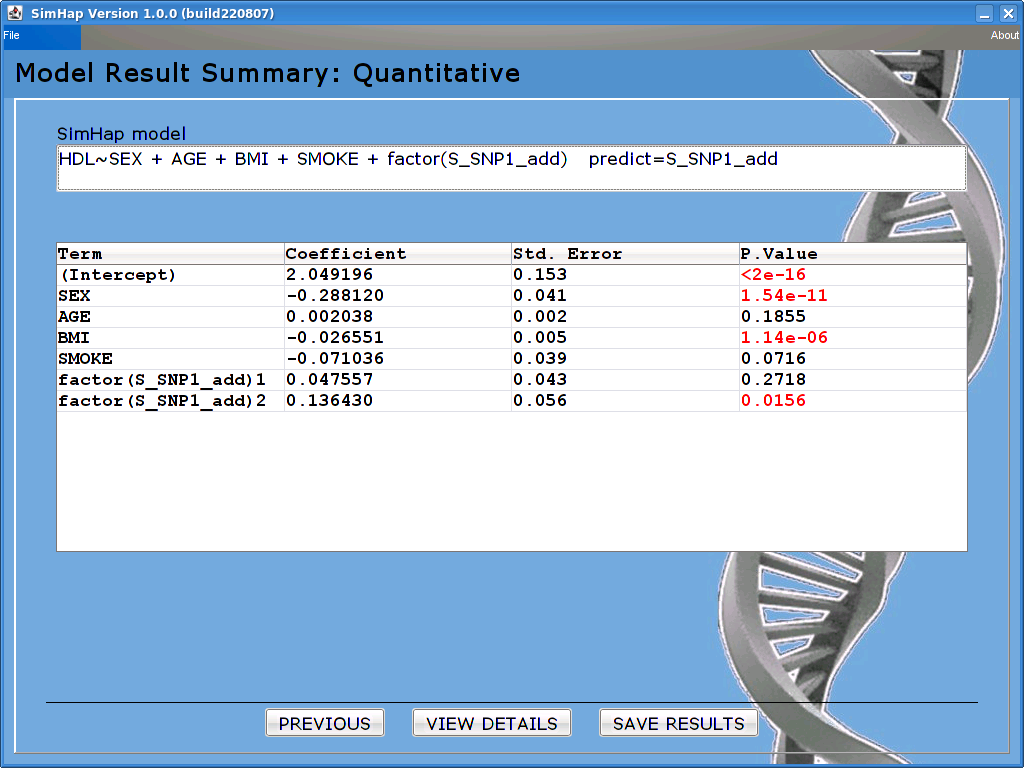

Supplement: Additional file 7 — SimHap GUI results summary. After the user has built their desired statistical model, SimHap GUI runs the analysis, and the summary results are presented as illustrated in this screenshot. Statistically significant results are highlighted in red for easy identification. [file 1471-2105-9-557-S7.png]

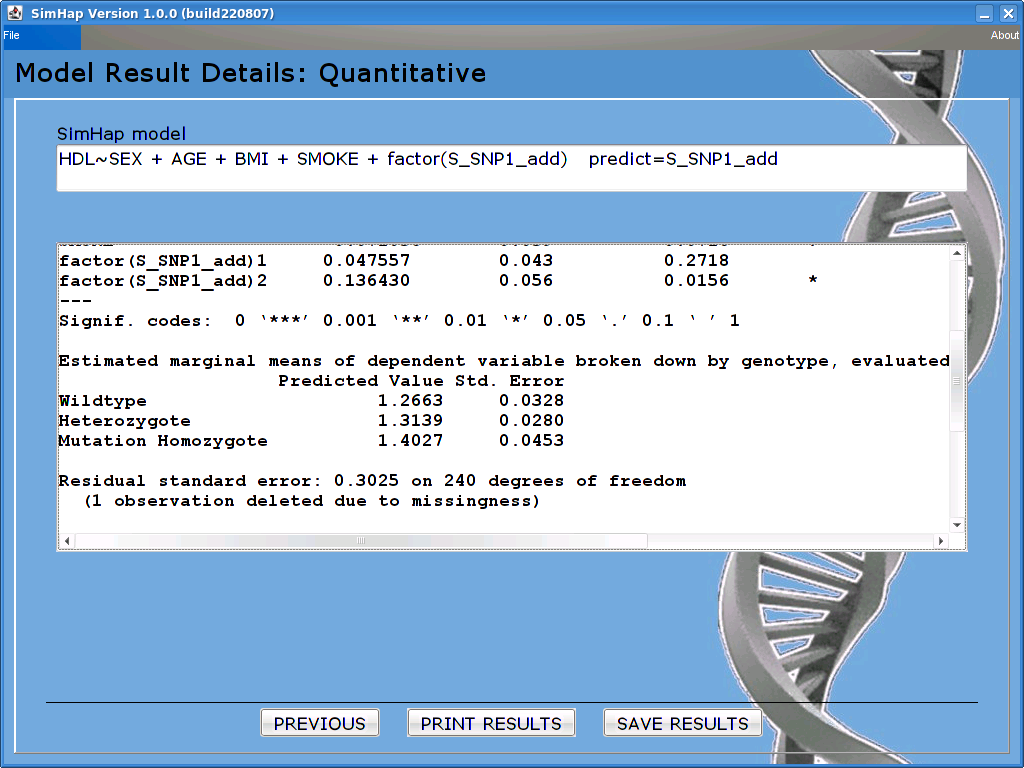

Supplement: Additional file 8 — SimHap GUI detailed results summary. The screenshot shows the detailed statistical information provided, in addition to the summary statistics described in the previous figure. For example, marginal means by genotype group are provided in this detailed summary. [file 1471-2105-9-557-S8.png]
